# *Pseudomonas pseudoalcaligenes* CECT5344, a cyanide-degrading bacterium with by-product (polyhydroxyalkanoates) formation capacity

**DOI:** 10.1186/s12934-015-0267-8

**Published:** 2015-06-10

**Authors:** Isabel Manso Cobos, María Isabel Ibáñez García, Fernando de la Peña Moreno, Lara Paloma Sáez Melero, Víctor Manuel Luque-Almagro, Francisco Castillo Rodríguez, María Dolores Roldán Ruiz, María Auxiliadora Prieto Jiménez, Conrado Moreno Vivián

**Affiliations:** Departamento de Bioquímica y Biología Molecular, Campus de Rabanales, Edificio Severo Ochoa, 1ª Planta Universidad de Córdoba, Córdoba, 14071 Spain; Departamento de Biología Medioambiental, Centro de Investigaciones Biológicas (CIB-CSIC), Madrid, Spain

**Keywords:** Alkalophile, Bioplastics, Cyanide degradation, Polyhydroxyalkanoates, *Pseudomonas*

## Abstract

**Background:**

Cyanide is one of the most toxic chemicals produced by anthropogenic activities like mining and jewelry industries, which generate wastewater residues with high concentrations of this compound. *Pseudomonas pseudoalcaligenes* CECT5344 is a model microorganism to be used in detoxification of industrial wastewaters containing not only free cyanide (CN^−^) but also cyano-derivatives, such as cyanate, nitriles and metal-cyanide complexes. Previous in silico analyses suggested the existence of genes putatively involved in metabolism of short chain length (scl-) and medium chain length (mcl-) polyhydroxyalkanoates (PHAs) located in three different clusters in the genome of this bacterium. PHAs are polyesters considered as an alternative of petroleum-based plastics. Strategies to optimize the bioremediation process in terms of reducing the cost of the production medium are required.

**Results:**

In this work, a biological treatment of the jewelry industry cyanide-rich wastewater coupled to PHAs production as by-product has been considered. The functionality of the *pha* genes from *P. pseudoalcaligenes* CECT5344 has been demonstrated. Mutant strains defective in each proposed PHA synthases coding genes (Mpha^−^, deleted in putative mcl-PHA synthases; Spha^−^, deleted in the putative scl-PHA synthase) were generated. The accumulation and monomer composition of scl- or mcl-PHAs in wild type and mutant strains were confirmed by gas chromatography-mass spectrometry (GC–MS). The production of PHAs as by-product while degrading cyanide from the jewelry industry wastewater was analyzed in batch reactor in each strain. The wild type and the mutant strains grew at similar rates when using octanoate as the carbon source and cyanide as the sole nitrogen source. When cyanide was depleted from the medium, both scl-PHAs and mcl-PHAs were detected in the wild-type strain, whereas scl-PHAs or mcl-PHAs were accumulated in Mpha^−^ and Spha^−^, respectively. The scl-PHAs were identified as homopolymers of 3-hydroxybutyrate and the mcl-PHAs were composed of 3-hydroxyoctanoate and 3-hydroxyhexanoate monomers.

**Conclusions:**

These results demonstrated, as proof of concept, that talented strains such as *P. pseudoalcaligenes* might be applied in bioremediation of industrial residues containing cyanide, while concomitantly generate by-products like polyhydroxyalkanoates. A customized optimization of the target bioremediation process is required to gain benefits of this type of approaches.

## Background

Cyanide is produced at high concentrations by anthropogenic sources like electroplating, jewelry and mining activities. Residues that contain high concentrations of cyanide must be remediated in order to remove cyanide, and biological approaches may display advantages over physicochemical treatments. The alkaliphilic bacterium *P. pseudoalcaligenes* CECT5344 is able to utilize cyanide and cyano-derivatives as the sole nitrogen source [1]. In the CECT5344 strain, cyanide induces various mechanisms for cyanide resistance and assimilation, such as cyanide-insensitive respiration [2], mechanisms for iron homeostasis [3] and synthesis of the specific nitrilase involved in the cyanide assimilation pathway [4]. Recently, the complete genome sequence of this bacterium has been reported [5, 6]. In addition to genes involved in cyanide assimilation and resistance, such as the *nit* genes encoding nitrilases and the *cio* genes coding for alternative cyanide-insensitive oxidases, this strain harbors some genes potentially involved in other processes with a great biotechnological potential, such as the production of polyhydroxyalkanoates (PHAs). The cost-efficient production of PHAs is averted partly due to the high costs of the carbon sources supplied to the production medium. Valorization and utilization of wastes via their bioconversion into bioplastics is one of the most distinctive strategies to unlock the PHAs production at industrial scale. In this sense, side production of PHAs as an extra-income (by-product) of non-strictly cost-dependent processes such as those driven to strategies for bioremediation of toxic compounds has not been extensively considered.

Many bacteria accumulate PHAs in the cytoplasm as carbon and energy storage material when growing under nutrient imbalanced conditions, but with an excess of a carbon source [7–10]. PHAs are biopolyesters that consist of 3-hydroxycarboxylic acids that are classified into two major groups displaying different material properties: the short chain length (scl-) with 3–5 carbon atoms or the medium chain length (mcl-) with 6–14 carbons [9–11]. Different proteins associated to the PHAs granules have been identified [11–15], such as PHA synthases for polymerization, PHA depolymerases involved in bioplastic degradation and monomer mobilization, and phasins with structural and regulatory functions [13, 16, 17]. Essential steps for PHAs biosynthesis are generation of the hydroxyacyl-CoA (HA-CoA) and the PHA synthase-catalyzed HA-CoAs polymerization into PHAs [18–20]. PHA synthases are classified into four types, of which class I and class II PHA synthases are composed of one subunit (PhaC). However, type I enzymes accept scl-HA-CoA for polymerization [21–28] whereas type II synthases, mainly found in pseudomonads, display substrate specificity towards mcl-HA-CoA [22]. Most pseudomonads produce polymers containing mcl-PHAs. However, a few strains like *Pseudomonas* sp. 61-3 [23], *Pseudomonas oleovorans* strain B-778 [24], *Pseudomonas pseudoalcaligenes* YS1 [25] and *Pseudomonas stutzeri* 1317 [26] synthesize a mixture of scl- and mcl-PHAs.

Three gene clusters putatively involved in the metabolism of scl- and mcl-PHAs have been identified in the genome of the cyanide-degrading bacterium *P. pseudoalcaligenes* CECT5344 [5]. The *phbRphaBAC* gene cluster includes the *phaB* gene that codes for an NADPH-dependent acetoacetyl coenzyme A reductase, the *phaA* gene encoding a β-ketothiolase, and the *phaC* gene coding for a class I scl-PHA synthase. The *phbR* gene shows similarity to members of the AraC/XylS transcriptional activators family. In the same locus, an additional cluster comprises the *phaP* and *phaR* genes that code for a phasin and a regulatory protein, respectively. In a different locus, a third gene cluster is similar to those found in other mcl-PHAs producers [revised in 10]. It comprises the *phaC1* and *phaC2* genes that code for class II PHA synthases, the regulatory *phaD* gene, the *phaF* and *phaI* genes encoding a phasin and a regulatory protein, respectively, and the *phaZ* gene, which is located between the *phaC1* and *phaC2* genes and encodes a putative depolymerase responsible for PHAs mobilization [29].

Cyanide degradation by the strain CECT5344 under alkaline conditions was previously optimized in a batch reactor loaded with a minimal medium containing acetate as carbon source and 2 mM NaCN as nitrogen source [30]. In this work we analyse the ability of this strain to bioremediate an industrial cyano-waste through a biological treatment that concomitantly generates PHAs as by-product.

## Results and discussion

### Cyanide assimilation and simultaneous synthesis of PHAs by *P. pseudoalcaligenes* CECT5344

**Figure 1:**
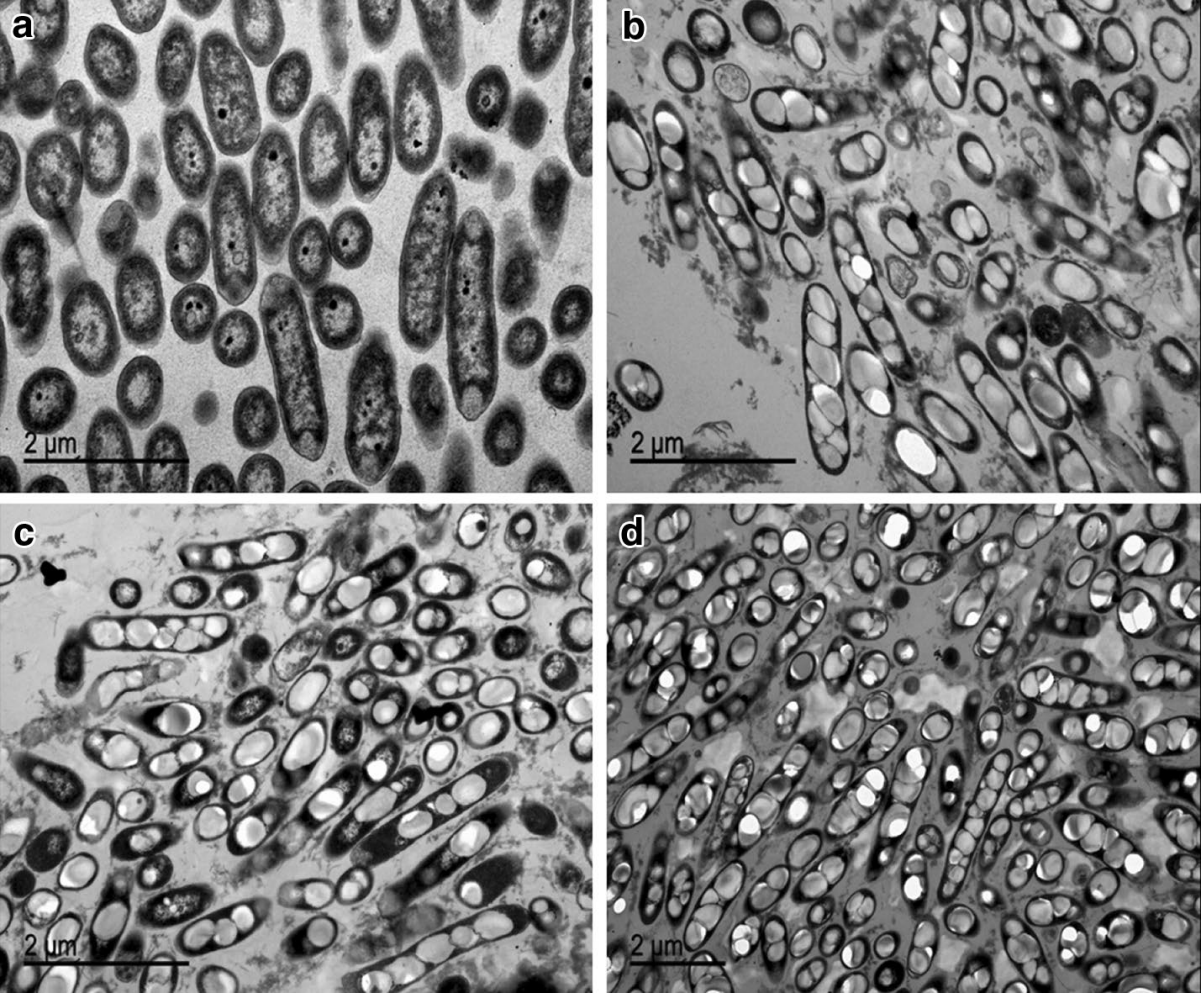
Detection by transmission electron microscopy of PHAs accumulated in *P. pseudoalcaligenes* CECT5344. Cells were grown with 50 mM sodium acetate and 2 mM ammonium chloride (**a**) or with 12.5 mM octanoate plus ammonium chloride (**b**), sodium cyanide (**c**), or cyanide from jewelry residue (**d**), each at 2 mM initial concentration. Samples were analyzed at 30 h of cultivation, before cyanide was completely consumed in cyanide-containing media.

Polyhydroxyalkanoates are reserve polyesters that are accumulated as intracellular granules in a variety of bacteria from a wide range of substrates such as renewable sources, sub-products, organic acids, fossil resources and wastes [31]. Nevertheless, in the bioremediation context, a limited number of microorganisms have been reported to accumulate PHAs from different toxic sources such as aromatic hydrocarbons [32–34], olive oil industry wastes [35] or textile dyes [36]. In a previous work, 2 mM NaCN added to a minimal medium was biologically detoxified by *P. pseudoalcaligenes* CECT5344 [30]. The main purpose of this work is to test the ability of the strain CECT5344 to accumulate PHAs during cyanide or industrial cyano-wastes assimilation, with the goal of providing the bases to pilot a bio-based treatment to bioremediate industrial cyano-wastes, yielding by-products. Firstly, to test the functionality of the predicted scl-PHA and mcl-PHA synthase genes identified in the genome of *P. pseudoalcaligenes* CECT5344 [5, 6], the ability of this strain to accumulate PHAs was tested in cells grown in Erlenmeyer flasks as described in Methods section, with media containing different carbon sources such as acetate or octanoate. PHAs accumulation was not detected in *P.pseudoalcaligenes* CECT5344 cells grown with 50 mM acetate and 2 mM ammonium (Figure 1a). Nevertheless, PHAs were accumulated in cells cultured with 2 mM ammonium and 12.5 mM octanoate, a suitable carbon substrate that supports cell growth and polymer production. This fact was confirmed qualitatively by transmission electron microscopy (Figure 1b). Similar PHAs accumulation was observed when this strain was grown with 12.5 mM octanoate as the C-source and either 2 mM sodium cyanide (Figure 1c) or with the jewelry wastewater, diluted to obtain a concentration of free cyanide of 2 mM, as the sole N-source (Figure 1d). In both media, cells were recovered when cyanide was not completely depleted proving that PHAs accumulation takes place in the presence of cyanide and it is not inhibited by this toxic compound. On the basis of these results, octanoate was chosen as the carbon source for further experiments.

A GC–MS analysis of methanolyzed samples of PHAs produced by *P. pseudoalcaligenes* CECT5344 was performed to determine the composition of the polymer produced. With this purpose, *P. pseudoalcaligenes* CECT5344 cells were cultured on shake flasks with 12.5 mM octanoate as carbon source and ammonium chloride, sodium cyanide or cyanide-containing jewelry waste, each added at 2 mM initial concentration, as the sole nitrogen source (Table 1). In this case, content and monomer composition of PHAs were determined when the nitrogen source was totally consumed (about 50 h with ammonium and near 100 h with cyanide). *P. pseudoalcaligenes* CECT5344 cells accumulated both scl-PHAs and mcl-PHAs in the three nitrogen sources tested, with the highest PHAs accumulation (about 85% of cell dry weight, CDW) in presence of ammonium. However, significant PHAs production was also achieved when sodium cyanide (47% of CDW) or the cyanide-rich industrial residue (55% of CDW) was used as the sole nitrogen source (Table 1). PHAs accumulation in the cells grown with the cyanide-containing jewelry waste was slightly higher than that observed with sodium cyanide as nitrogen source (Table 1). This difference could be due to the presence of iron in the jewelry waste, since this metal has significant influence on PHA synthesis. Mohan and Reddy [37] demonstrated that a high iron concentration in the media increases PHAs production by mixed cultures. They also proposed that iron can interact with other parameters or factors, such as pH, nitrogen, phosphorous concentration or C-source composition, to maximize PHA production [37]. Under all these culture conditions, the mcl-PHAs accumulated by *P. pseudoalcaligenes* CECT5344 contained about 95% 3-hydroxyoctanoate (OH-C8) and 5% 3-hydroxyhexanoate (OH-C6) monomers (Table 1), as described for other pseudomonads when cultured in octanoic acid [10]. The scl-PHAs accumulated were found to be homopolymers of 3-hydroxybutyrate. The mcl-PHAs content detected in cells grown with the jewelry waste was slightly higher (26% of CDW) than that assessed when the nitrogen source was ammonium (20% of CDW) or sodium cyanide (16% of CDW). However, the scl-PHAs content in cells grown with both cyanide-containing media was similar (about 30% of CDW) (Table 1). These results demonstrate the functionality of the scl-PHA and mcl-PHA synthase genes from *P. pseudoalcaligenes* CECT5344, in both ammonium and cyanide-containing media.

**Table 1:** PHAs accumulation in the wild-type and PHA synthase defective mutants of *P. pseudoalcaligenes* CECT5344

Nitrogen source	Strain	CDW (g L^−1^)	PHAs content			
			scl-PHAs (% CDW)	mcl-PHAs (% CDW)	mcl-PHA monomer composition (%)	
					OH-C6	OH-C8
Ammonium chloride	Wild-type	1.18 ± 0.1	64.95 ± 3.60	20.12 ± 0.08	5.75 ± 0.49	94.25 ± 0.35
	Mpha^−^	0.76 ± 0.05	66.14 ± 1.95	–	–	–
	Spha^−^	0.48 ± 0.03	–	36.81 ± 1.90	9.16 ± 0.08	90.84 ± 0.10
	Mpha^−^/Spha^−^	0.34 ± 0.03	–	–	–	–
Sodium cyanide	Wild-type	0.22 ± 0.04	31.02 ± 3.25	16.37 ± 2.81	5.97 ± 0.11	94.03 ± 0.14
	Mpha^−^	0.28 ± 0.02	55.47 ± 4.29	–	–	–
	Spha^−^	0.18 ± 0.02	–	15.44 ± 1.05	6.08 ± 0.08	93.92 ± 0.06
	Mpha^−^/Spha^−^	0.22 ± 0.03	–	–	–	–
Jewelry residue	Wild-type	0.34 ± 0.03	29.29 ± 0.74	25.63 ± 1.27	5.58 ± 0.07	94.42 ± 0.05
	Mpha^−^	0.36 ± 0.04	39.92 ± 2.12	–	–	–
	Spha^−^	0.28 ± 0.04	–	19.94 ± 0.02	5.43 ± 0.06	94.57 ± 0.13
	Mpha^−^/Spha^−^	0.28 ± 0.05	–	–	–	–

Cells were cultured in Erlenmeyer-flasks containing minimal medium with 12.5 mM octanoate and ammonium chloride, sodium cyanide or cyanide from the jewelry residue (2 mM each), and measurements were performed when the N source was totally consumed. Data correspond to the media of three measurements.

*CDW* cell dry weight, *OH-C6* 3-hydroxyhexanoate, *OH-C8* 3-hydroxyoctanoate.

### By-product accumulation by *P. pseudoalcaligenes* CECT5344 under cyanide detoxification culture conditions

**Figure 2:**
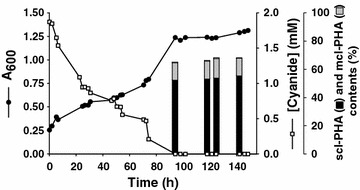
Bacterial growth, cyanide consumption and PHAs accumulation in a batch reactor culture of *P. pseudoalcaligenes* CECT5344. Experiment was conducted in a bioreactor with 12.5 mM octanoate as carbon source and 2 mM cyanide from the jewelry residue as the sole nitrogen source, under conditions described in Methods. Cell growth was monitored by estimating the absorbance at 600 nm (A_600_). At the indicated times, cells were collected by centrifugation and the PHAs content was analyzed by GC–MS. The scl-PHAs content (*black bars*) and the mcl-PHAs content (*grey bars*) are given as percentage of CDW.

The strain CECT5344 is able to accumulate both scl- and mcl-PHAs when sodium cyanide or the jewelry residue is used as the sole nitrogen source, and octanoate as carbon source, in flask cultures (Table 1). However, the ability of *P. pseudoalcaligenes* CECT5344 to produce PHAs in batch reactor under cyanide degradation conditions with the cyanide-rich jewelry residue as nitrogen source need to be verified. Accumulation of PHAs was tested in a 5 L reactor with M9 minimal media using 12.5 mM octanoate and the cyanide-containing wastewater (2 mM free cyanide) as carbon and nitrogen sources, respectively, under the operational conditions described in Methods section. Cell growth (A_600_), cyanide assimilation and PHAs accumulation (monitored by GC–MS) were followed for 145 h (Figure 2). The production of PHAs was determined after 90 h, when cyanide was completely consumed and about 10% octanoate (1.2 mM) was still remaining in the media. As deduced from the GC–MS analysis, the total PHAs content in wild-type strain was about 66% of CDW, with 53% scl-PHAs and 13% mcl-PHAs (Figure 2). The monomer composition of the PHAs was similar to that observed in flasks cultures (Table 1). Other strains like *Pseudomonas* 3Y2 produce PHAs consisting of both scl- and mcl-HA units, with 10–30% of scl-PHAs when growing on gluconate, octanoate, dodecanoate or oleate. However, *Pseudomonas* 3Y2 has only two PHA synthases belonging to class II, which display broad substrate specificities and are capable of catalyzing the incorporation of scl-PHA as well as mcl-3HA units (C6-C12) into PHA polymers [38], lacking genes for specific scl-PHA metabolism. *P. pseudoalcaligenes* YS1 also produces both scl- and mcl-PHAs from octanoic and myristic acids, but only a PHA synthesis pathway responsible for production of mcl-PHAs was identified [25].

### Broadening the portfolio of PHA by-products from cyano-wastes

**Figure 3:**
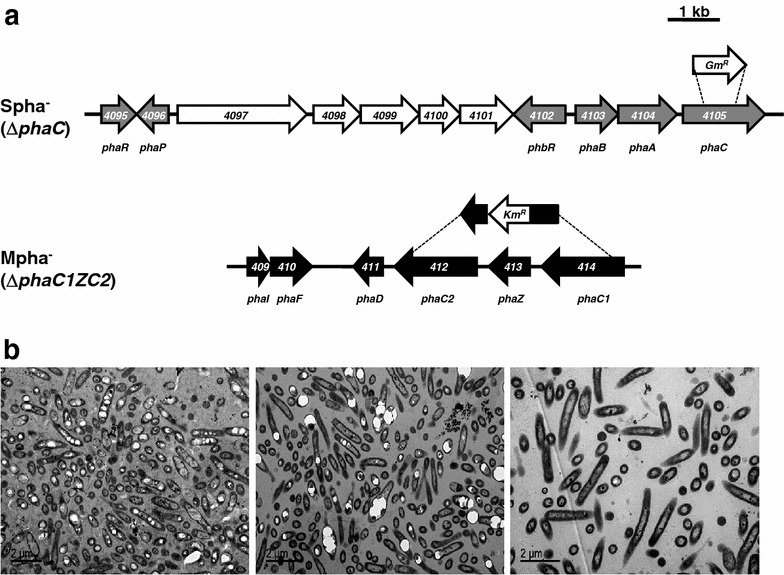
PHAs accumulation in *P. pseudoalcaligenes* CECT5344 mutant strains defective in PHA synthesis. **a** Strategy followed for generation of the Mpha^−^ and Spha^−^ mutants of *P. pseudoalcaligenes* CECT5344. **b** Transmission electron microscopy images of Mpha^−^ (*left*), Spha^−^ (*middle*) and double Mpha^−^/Spha^−^ (*right*) mutant strains of *P. pseudoalcaligenes* CECT5344. Cells were grown with 12.5 mM sodium octanoate and the cyanide-containing residue from the jewelry industry (2 mM initial concentration). Samples were analyzed at 30 h of cultivation, before cyanide was completely consumed.

To further control the type of by-product accumulated, three strains of *P. pseudoalcaligenes* CECT5344 defective in PHA synthases (Mpha^−^, Spha^−^ and double Mpha^−^/Spha^−^ mutants) were constructed (Figure 3a). The mutant strain Mpha^−^ was constructed by deletion of the *phaC1ZC2* genes and insertion of a kanamycin resistance gene. The mutant Spha^−^ was generated by disruption of *phaC* gene and insertion of a gentamicin resistance cassette. As control strain, the double Mpha^−^/Spha^−^ mutant was constructed by disruptions of both *phaC1ZC2* and *phaC* genes. Figure 3b shows the transmission electron microscopy images of Mpha^−^, Spha^−^ and Mpha^−^/Spha^−^ mutants grown with 12.5 mM octanoate as carbon source and the jewelry residue containing 2 mM cyanide as nitrogen source. PHAs accumulation in Mpha^−^ (Figure 3b, left) or Spha^−^ (Figure 3b, middle) mutants was less evident than in wild-type cells grown with jewelry residue (Figure 1d). PHAs accumulation was not detected in the Mpha^−^/Spha^−^ double mutant (Figure 3b, right).

The ability of these mutant strains to accumulate PHAs was also tested in batch cultures on shake flasks with 12.5 mM octanoate as carbon source and either ammonium chloride, sodium cyanide or cyanide from the jewelry residue, each at 2 mM initial concentration, as the sole nitrogen source (Table 1). The PHA type accumulated by *P. pseudoalcaligenes* CECT5344 during degradation of the cyanide-containing industrial residue has been found to be different depending on the bacterial strain used; scl-PHAs (Mpha^−^ mutant), mcl-PHAs (Spha^−^ mutant) or a mixture of both scl- and mcl-PHAs (wild-type). By contrast to wild-type, the Mpha^−^ strain defective in the *phaC1ZC2* genes only accumulated scl-PHAs, whereas the Spha^−^ strain deficient in the *phaC* gene only synthesized mcl-PHAs. The Mpha^−^/Spha^−^ double mutant was unable to accumulate PHAs. The scl-PHAs content in Mpha^−^ cells cultured with sodium cyanide was higher than in cells grown with jewelry residue, but the mcl-PHAs content in Spha^−^ cells was lower with NaCN than in the presence of the cyanide-containing residue (Table 1). The scl-PHAs obtained in the Mpha^−^ mutant were homopolymers of 3-hydroxybutyrate whereas the mcl-PHA monomer composition accumulated by Spha^−^ mutant was similar to those observed in wild type cells, with about 95% 3-hydroxyoctanoate and 5% 3-hydroxyhexanoate monomers (Table 1). Although PHAs content of cyanide-grown cells was not very high for an industrial purpose by itself, it may be attractive as a value-added of the cyanide detoxification process. It is worth to mention that the jewelry industry located in the city of Córdoba, Spain, produces 4–5 tons per year of an alkaline residue containing up to 26 g L^−1^ of free cyanide (around 1 M) together with high amounts of heavy metals [1], thus making this effluent highly toxic and environmentally hazardous.

**Figure 4:**
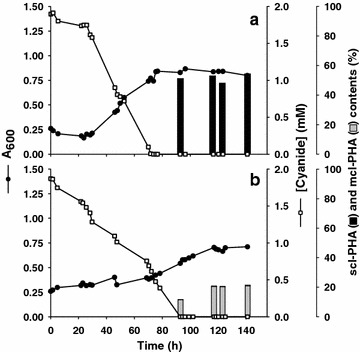
Bacterial growth, cyanide consumption and PHAs accumulation in batch reactor cultures of the Mpha^−^ (**a**) and Spha^−^ (**b**) mutant strains. Experiments were conducted in a 5 L bioreactor with 12.5 mM octanoate as carbon source and 2 mM cyanide from the jewelry residue as the sole nitrogen source, under conditions described in Methods. Bacterial growth was monitored by estimating the absorbance at 600 nm (A_600_). At the indicated times, cells were collected by centrifugation and the PHAs content was analyzed by GC–MS. The scl-PHAs content (*black bars*) and the mcl-PHAs content (*grey bars*) are given as percentage of CDW.

The ability of *P. pseudoalcaligenes* mutant strains to accumulate PHAs in batch reactor with the cyanide-rich jewelry residue as nitrogen source was also analyzed by the same procedure described for the wild-type strain. As expected, there were no significant differences in the ability to assimilate cyanide and octanoate between wild-type and mutant strains. After 90 h cyanide was completely consumed in all cultures and less than 10% octanoate remained in the media (Figures 2, 4). The amount of scl-PHAs accumulated in the Mpha^−^ mutant was about 53% of CDW (Figure 4a), similar to that observed for the wild-type strain (Figure 2). The mcl-PHAs content in the Spha^−^ mutant was about 21% of CDW (Figure 4b), slightly higher than in the wild-type strain (Figure 2). However, depending on the cultivation procedure, flask or reactor, the percentage of scl-PHAs and mcl-PHAs slightly changed in both wild-type and Mpha^−^ mutant cells (Table 1; Figures 2, 4). It is worth to mention that in bioreactor cultures both pH (9.5) and oxygen saturation (10%) were kept constant throughout the bioremediation process, which is not the case in flask cultures.

### Phylogenetic analysis of the *P. pseudoalcaligenes* CECT5344 PHA synthases

**Figure 5:**
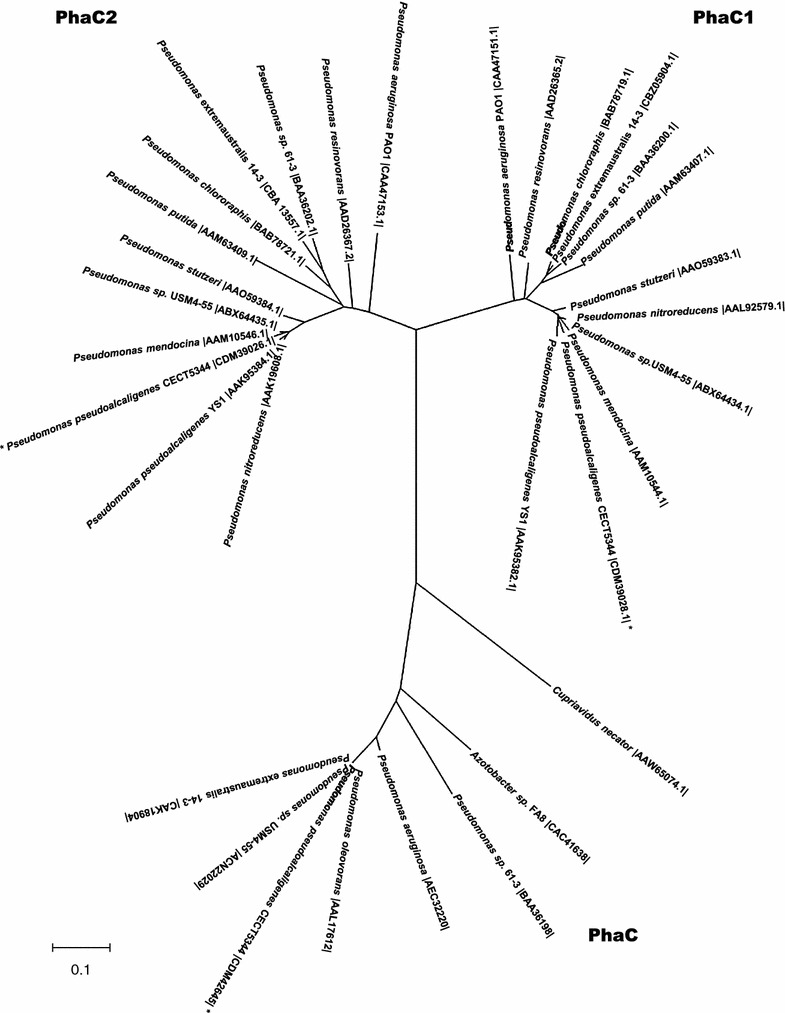
Phylogenetic tree of PHA synthases. The evolutionary history was inferred using the Neighbor-Joining method [54]. The optimal tree with the sum of branch length = 2.80718513 is shown. The tree is drawn to scale, with branch lengths in the same units as those of the evolutionary distances used to infer the phylogenetic tree. The evolutionary distances were computed using the Poisson correction method [55] and are in the units of the number of amino acid substitutions per site. The analysis involved 32 amino acid sequences. All positions containing gaps and missing data were eliminated. There were a total of 294 positions in the final dataset. Evolutionary analyses were conducted in MEGA5 [56]. Sequences for PhaC1 synthases: *P. aeruginosa* PAO1 [GenBank:CAA47151.1], *P. resinovorans* NRRL B-2649 [GenBank:AAD26365.2], *P. chlororaphis* subsp. *aureofaciens* [GenBank:BAB78719.1], *P. extremaustralis* 14-3 [GenBank:CBZ05904.1], *Pseudomonas* sp. 61-3 [GenBank:BAA36200.1], *P. putida* KT2440 [GenBank:AAM63407.1], *P. stutzeri* 1317 [GenBank:AAO59383.1], *P. nitroreducens* [GenBank:AAL92579.1], *Pseudomonas* sp. USM4-55 [GenBank:ABX64434.1], *P. mendocina* ymp [GenBank:AAM10544.1], *P. pseudoalcaligenes* CECT5344 [GenBank:CDM39028.1] and *P. pseudoalcaligenes* YS1 [GenBank:AAK95382.1]. Sequences for PhaC2 synthases: *P. aeruginosa* PAO1 [GenBank:CAA47153.1], *P. resinovorans* NRRL B-2649 [GenBank:AAD26367.2], *Pseudomonas* sp. 61-3 [GenBank:BAA36202.1], *P. extremaustralis* 14-3 [GenBank:CBA13557.1], *P. chlororaphis* subsp. *aureofaciens* [GenBank:BAB78721.1], *P. putida* KT2440 [GenBank:AAM63409.1], *P. stutzeri* 1317 [GenBank:AAO59384.1], *Pseudomonas* sp. USM4-55 [GenBank:ABX64435.1], *P. mendocina* ymp [GenBank:AAM10546.1], *P. pseudoalcaligenes* CECT5344 [GenBank:CDM39026.1], *P. pseudoalcaligenes* YS1 [GenBank:AAK95384.1] and *P. nitroreducens* [GenBank:AAK19608.1]. Sequences for PhaC synthases: *Cupriavidus necator* VKPM B5786 [GenBank:AAW65074.1], *Azotobacter* sp. FA8 [GenBank:CAC41638], *Pseudomonas* sp. 61-3 [GenBank:BAA36198], *P. aeruginosa* DM2 [GenBank:AEC32220], *P. oleovorans* NRRL B-778 [GenBank:AAL17612], *P. pseudoalcaligenes* CECT5344 [GenBank:CDM42645], *Pseudomonas* sp. USM4-55 [GenBank:ACN22029] and *P. extremaustralis* 14-3 [GenBank:CAK18904].

The genome of *P. pseudoalcaligenes* CECT5344 harbors three putative PHA synthase genes involved in metabolism of scl-PHAs (*phaC* gene) and mcl-PHAs (*phaC1* and *phaC2* genes) located in three different clusters (Figure 3a). The PhaC1 and PhaC2 synthases involved in mcl-PHAs and the PhaC synthase for scl-PHAs are rarely found together in other bacterial strains, excluding some pseudomonads like the *Pseudomonas* strains 61-3, USM 4-55 and 3Y2 (Figure 5). Thus, the *phbRphaBAC* and the *phaC1ZC2D* gene clusters have been identified in *Pseudomonas* sp. strain 61-3 and *Pseudomonas* sp. USM 4-55, respectively [23, 39]. The class I synthase for scl-PHAs (PhaC) and the two synthases of type II for mcl-PHAs (PhaC1 and PhaC2) of these *Pseudomonas* strains show homology to the corresponding synthases found in the strain CECT5344 (Figure 5). *P. extremaustralis* 14-3 also contains both *phaC* and *phaC1ZC2* homologues genes, although the *phaI* and *phaF* genes are not located downstream the *phaC1ZC2D* genes [40]. The PhaC1 and PhaC2 synthases of *P. pseudoalcaligenes* CECT5344 share 57% identity and belong to class II PHA synthases, whereas the *P. pseudoalcaligenes* CECT5344 PhaC synthase is a member of class I PHA synthases and shares 52% identity with PhaC of *Cupriavidus necator*. Some *Pseudomonas* strains that contain homologues to *P. pseudoalcaligenes phaC1* and *phaC2* genes, but lack the *phaC* gene homologue, are *P. mendocina* ymp, *P. stutzeri* 1317*, P. putida* KT2440*, P. resinovorans* NRRL B-2649 and *P. nitroreducens* 0802 (Figure 5). All these *Pseudomonas* species harbor the *phaC1ZC2DFI* gene cluster for mcl-PHAs metabolism. On the other hand, only a few strains of *Pseudomonas* have been reported to possess the *phaC* gene encoding a type I synthase for scl-PHAs (Figure 5). *Pseudomonas stutzeri* A1501 contains exclusively a *phaC* gene homologue, which exhibits 100% identity with *P. pseudoalcaligenes* CECT5344 *phaC* gene, but there are not available data about the type of polymer synthesized by this strain [41].

The *phbRphaBAC* gene cluster of *P. pseudoalcaligenes* CECT5344 shows similar gene arrangement to those found in other bacteria, like *Pseudomonas* sp. 61-3 [23], *Pseudomonas* sp. USM 4-55 [42], *Azotobacter* sp. FA8 [43] and *Azotobacter vinelandii* [44]. In *P. extremaustralis* 14-3 the *phbRphaBAC* gene cluster is located within the *pha*GI genomic island, which is flanked by an 8 bp direct repeat sequence, 5′-TTTTTTGA-3′, and shares strong similarity with the genomic islands found in diverse *Proteobacteria*, including *Azotobacter vinelandii* and *Burkholderiales* species [45]. The G + C content, phylogeny inference and codon usage analysis show that the *phaBAC* genes have a complex mosaic structure and suggest that *phaB* and *phaC* genes could be acquired by horizontal gene transfer, probably from *Burkholderiales* [45]. The *P. pseudoalcaligenes* CECT5344 *phbRphaBAC* gene cluster might be also acquired by horizontal gene transfer, although it lacks the direct repeat sequence and the composition of the neighbor genes is also different.

## Conclusions

Our results demonstrate that the cyanotrophic bacterium *P. pseudoalcaligenes* CECT5344 is able to carry out the biodegradation of toxic cyanide-rich jewelry wastewater associated with production of PHAs as by-product. Experiments performed in flasks or in bioreactor cultures support that the type of PHA accumulated can be tailored. Both Mpha^−^ (mcl-PHAs minus) and Spha^−^ (scl-PHAs minus) mutants are able to assimilate cyanide, as well as wild-type, but Mpha^−^ strain produces exclusively scl-PHAs and Spha^−^ only produces mcl-PHAs, whereas the wild-type strain accumulates both types of PHAs. This constitutes a proof of concept to design more profitable processes to biodegrade cyanide-containing wastes.

## Methods

### Bacterial strains, growth conditions and plamids

*P. pseudoalcaligenes* CECT5344 (CECT: Spanish Type Culture Collection) was isolated by cyanide-enrichment cultivation from samples of the Guadalquivir River (Córdoba, Spain). This strain was able to assimilate cyanide as sole nitrogen source under alkaline conditions and it was classified as *Pseudomonas pseudoalcaligenes* by its 16S RNA sequence analysis [1]. The bacterial strains of *Escherichia coli* and *P. pseudoalcaligenes* were grown in LB rich medium [46] at 37 and 30°C, respectively. The appropriate antibiotics, nalidixic acid (10 μg mL^−1^), ampicillin (150 μg mL^−1^), gentamicin (20 μg mL^−1^) and kanamycin (25 μg mL^−1^), were added when required.

For the analysis of PHAs production in flask cultures, *P. pseudoalcaligenes* CECT5344 was first grown in LB media in order to obtain a large biomass. These cultures were centrifuged and the cells were suspended in M9 minimal medium [46] as a source of inoculums. The media were adjusted to pH 9.5 and inoculated with the appropriate cell amount to reach an initial absorbance at 600 nm (A_600_) of about 0.1. Cells were grown in 500-mL Erlenmeyer flasks containing 200 mL of M9 minimal media with 12.5 mM octanoate, but when indicated, sodium acetate (50 mM) was also used as the sole carbon source. Ammonium chloride, sodium cyanide or cyanide-containing jewelry residue (2 mM initial concentration) was added as the sole nitrogen source. Therefore, in all media, a C/N ratio of 50/1 was used. The jewelry industry residue, which contains cyanide in addition to metals like iron, copper and zinc, was supplied by the companies GEMASUR S.L. and SAVECO S.L., which handle the industrial residues from Córdoba.

### Batch reactor culture conditions

Experiments were carried out in a Biostat® B plus (Sartorius BBI Systems) 5 L bioreactor, using the following operational procedure based on [30]. The reactor was loaded with M9 minimal medium and further autoclaved. The working volume was 5 L. Sodium octanoate (12.5 mM) and jewelry residue (2 mM cyanide) were used as carbon and nitrogen sources, respectively. MgSO_4_ and FeSO_4_ solutions were sterilized by filtration and added to the M9 trace solution after autoclaving. Antifoam and the appropriate antibiotics were also added to the media. The reactor was then inoculated with the appropriate bacterial strain to reach an initial A_600_ of about 0.2 (25 ml of a bacterial suspension obtained from an overnight 500 ml LB culture). Temperature was maintained at 30°C and pH was initially adjusted to 9.5 and kept constant by automatic addition of 1 M NaOH. Continuous agitation at 450 rpm and dissolved oxygen saturation at 10% were controlled automatically. To prevent cyanhydric acid (HCN) losses, a bioreactor exhaust cooler was connected to a washing flask containing a concentrated NaOH solution. The absence of cyanide in samples from this flask was confirmed during the process.

### Analytical determinations

Cell growth was determined by following absorbance at 600 nm. To estimate biomass calculation, cell densities (expressed in grams of CDW per liter) were determined gravimetrically by using 50 mL Falcon tubes. Ammonium concentration was determined by the Nessler reagent as previously described [47]. Cyanide concentration was determined colorimetrically [48].

### DNA manipulations and generation of mutant strains

DNA manipulations and other molecular biology techniques were essentially performed as described previously [46]. The mutant strain Mpha^−^ of *P. pseudoalcaligenes* was constructed by deletion of the *phaC1ZC2* genes and insertion of a kanamycin cassette resistance gene. In this mutant, deletion of both *phaC1* and *phaC2* genes was partial, whereas *phaZ* gene was completely deleted. A central region of 619 bp in the *phaC1* gene was cloned into pBluescript II KS (±) after PCR amplification with primers phaC1E (5′-GAAGCCTTCGAATTCGGCAAGAAC-3′; *Eco*RI restriction site is underlined) and phaC1S (5′-GCAGGTAGTTGTCGACCCAGTAGTTC-3′; *Sal*I restriction site is underlined) to yield the pBKS-A construct. An internal fragment of 506 bp of the *phaC2* gene was amplified by PCR using genomic DNA from *P. pseudoalcaligenes* CECT5344 as template and the oligonucleotides phaC2S (5′-TCGAAGTCGACCGCAATCTGG-3′; *Sal*I restriction site is underlined) and phaC2X (5′-CTTGGCTGACTCGAGGGTTTCC-3′; *Xho*I restriction site is underlined). This fragment was digested with the appropriate restriction enzymes and cloned into the unique *Sal*I and *Xho*I sites of the pBKS-A plasmid to yield pBKS-AB. The 2.2 kb *Xho*I/*Sal*I fragment that contains the kanamycin cassette resistance gene from pSUP2025 [49] was ligated into the pBKS-AB vector previously digested with *Sal*I to generate pBKS-AKB. This construct was digested with *Eco*RI and *Kpn*I, yielding a 3.3 kb fragment that contains the kanamycin cassette resistance gene flanked by the internal region of the *phaC1* and *phaC2* genes. The resulting fragment was cloned into pK18mob, a suicide plasmid for *Pseudomonas* that confers kanamycin resistance but lacks of a functional replication origin for this bacterium [50]. The final construct pK18mob-AKB was used to deliver the Δ*phaC1ZC2* mutation to the host chromosome via homologous recombination. Biparental mating was performed using *E. coli* S17-1 (pK18mob-AKB) as the donor strain and a spontaneous nalidixic acid resistant mutant of *P. pseudoalcaligenes* CECT5344 as the recipient strain. Transconjugants were selected in M9 media with nalidixic acid and kanamycin. Disruption of *phaC1ZC2* genes was confirmed by PCR sequencing analysis.

To generate the Spha^−^ mutant of *P. pseudoalcaligenes*, the *phaC* gene was amplified by PCR using the oligonucleotides phbB (5′-GCCTGCTGCACGAGATCTTCCGCC-3′; *Bgl*II restriction site is underlined) and phbH (5′-ATTGGCGCTGGCGAAGCTTGAACCC-3′; *Hin*dIII restriction site is underlined) and *P. pseudoalcaligenes* CECT5344 genomic DNA as template. The amplified DNA fragment was then inserted into pBluescript II KS (±) previously digested with *Eco*RV and *Sma*I to generate the pBKS-*phaC* construct. The 1 kb *Eco*RI/*Pst*I fragment that contains the gentamicin resistance cassette from pMS255 [51] was ligated into the pBKS-*phaC* vector to generate the pBKS-Δ*phaC* construct. The restriction enzymes *Spe*I and *Hin*dII were used to digest pBKS-Δ*phaC*, generating a 2.1 kb fragment containing the *phaC* gene disrupted by the gentamicin resistance cassette. This fragment was then ligated into the pK18mob vector previously digested with *Hin*dIII and *Xba*I to generate pK18mob-Δ*phaC*. Biparental mating was performed using *E. coli* S17-1 (pK18mob-Δ*phaC*) as donor strain and the nalidixic acid resistant mutant of *P. pseudoalcaligenes* CECT5344 as recipient strain. Transconjugants (nalidixic acid and gentamicin resistant, and kanamycin sensitive) were isolated. Disruption of *phaC* gene was confirmed by PCR sequencing.

A double mutant Mpha^−^/Spha^−^ was obtained by biparental mating using *E. coli* S17-1 (pK18mob-Δ*phaC*) as donor strain and the *P. pseudoalcaligenes* Mpha^−^ mutant as recipient strain. Tranconjugants were selected in M9 with nalidixic acid, kanamycin and gentamicin. Disruptions of *phaC1ZC2* and *phaC* genes were confirmed by PCR sequencing.

### Transmission electron microscopy

Cells were harvested, washed twice in M9 minimal medium and fixed in 2% (w/v) glutaraldehyde in the same solution. Then, cells were suspended in 1% (w/v) OsO_4_ for 1 h, gradually dehydrated in acetone 30, 50, 70, 90 and 100% (v/v), 30 min each, and finally treated with propylene oxide (two changes, 10 min each). Afterwards, cells were embedded sequentially into 2:1, 1:1, 1:2 propylene oxide-resin. Ultrathin sections (thickness 50 nm) were cut with a Leica Ultracut R ultramicrotome (Leica Inc, Buffalo, USA) using a diatome diamond knife. The sections were picked up with 200 mesh cupper grids coated with a layer of carbon and subsequently observed in a Jeol JEM-1400 (Tokyo, Japan) electron microscope. These analyses were carried out by using the microscopy facilities at the central services for research support (SCAI) of the University of Córdoba (Spain).

### Gas chromatography-mass spectrometry (GC–MS) analysis for PHAs and octanoate determinations

Polyhydroxyalkanoate monomer composition and cellular PHAs content were determined by GC–MS of the methanolysed polyester. Samples of 50–150 mL culture medium were centrifuged for 20 min at 12,000×*g* and 4°C. Cell pellets were freeze-dried for 24 h in a lyophilizer and weighed. Methanolysis procedure was carried out by suspending 5–10 mg of lyophilized aliquots in 0.5 mL chloroform and 2 mL methanol containing 15% sulfuric acid and 0.5 mg mL^−1^ 3-methylbenzoic acid (internal standard) and then incubated at 80°C for 7 h. After cooling, 1 mL demineralized water and 1 mL chloroform were added, and the organic phase containing the resulting methyl esters of monomers was analyzed by GC–MS [52]. An Agilent series 7890A coupled with 5975C MS detector (EI, 70 eV) and a split-splitless injector were used for analysis. An aliquot (1 µL) of organic phase was injected into the gas chromatograph at a split ratio 1:20. Separation of compounds was achieved using an HP-5 MS capillary column (5% phenyl-95% methyl siloxane, 30 m × 0.25 mm film thickness). Helium was used as carrier gas at a flow rate of 1 mL min^−1^. The injector and transfer line temperature were set at 275 and 300°C, respectively. Oven temperature was initially 80°C for 2 min, then rose from 80°C up to 150°C at 5°C min^−1^, and kept at 150°C for 1 min. The mass spectra were recorded in full scan mode (m/z 40–550). 3-hydroxybutyric acid methyl ester was resolved using selected ion monitoring mode (SIM).

Octanoate concentration in the medium was analyzed by GC–MS following the procedure described by Escapa et al. [53].
